# Identification of Enhancers and Transcription Factors Regulating Postnatal Growth of Skeletal Muscle in Cattle

**DOI:** 10.3390/biology15151248

**Published:** 2026-07-29

**Authors:** Pengcheng Lyu, Binod Pokhrel, Jianguo Zhao, Honglin Jiang

**Affiliations:** 1 State Key Laboratory of Organ Regeneration and Reconstruction, Institute of Zoology, Chinese Academy of Sciences, Beijing 100101, China; pengcheng@vt.edu; 2 Institute for Stem Cell and Regeneration, Chinese Academy of Sciences, Beijing 100049, China; 3 School of Animal Sciences, Virginia Tech, Blacksburg, VA 24061, USA; drbinod@vt.edu

**Keywords:** cattle, enhancer, motif, skeletal muscle, transcription factor

## Abstract

Skeletal muscle undergoes extensive developmental and functional changes during the transition from neonatal to adult stages, but the biological processes and regulatory mechanisms underlying these changes remain incompletely understood. In this study, we integrated RNA-seq and H3K27ac ChIP-seq analyses to characterize transcriptional and epigenomic changes during muscle maturation in neonatal and adult Angus-cross cattle. RNA-seq analysis of longissimus muscle identified 2785 differentially expressed genes (DEGs) between neonatal and adult muscles. Adult muscle showed increased expression of genes associated with muscle maturation, whereas neonatal muscle exhibited higher activity of pathways related to energy metabolism and protein translation. H3K27ac profiling revealed extensive remodeling of active regulatory elements during muscle maturation, including stage-specific enhancers and super-enhancers. Motif enrichment analysis indicated distinct transcription factor networks associated with neonatal- and adult-specific regulatory landscapes. Neonatal muscle-specific enhancers were enriched for motifs of myogenic regulators, including MYOD1, MYOG, SIX family members, and ASCL2, whereas adult muscle-specific enhancers showed increased enrichment of the MEF2 family members. Integration of transcriptomic and epigenomic datasets identified stage-specific enhancers associated with genes involved in neonatal and adult muscle functions. Together, these findings demonstrate that the transition from neonatal to adult muscle is accompanied by coordinated changes in transcriptional programs and enhancer landscapes, providing new insights into the regulatory mechanisms underlying skeletal muscle maturation.

## Simple Summary

Skeletal muscle undergoes significant changes after birth, but the biological processes and regulatory mechanisms responsible for these changes are not fully understood. Understanding how muscle develops and grows from birth to adulthood in cattle is important for improving production and quality of beef, which is an important source of food for humans. In this study, we compared gene expression profiles and regulatory mechanisms in skeletal muscle between newborn calves and adult steers. Our results indicate that from birth to adulthood, biological processes in skeletal muscle shift from increased protein translation and energy metabolism to increased cell differentiation and migration, angiogenesis, fibrogenesis, and immune response. Our results further indicate that these biological changes are driven by coordinated shifts in transcription factor networks and enhancer-mediated regulatory programs. These results provide new insights into how skeletal muscle develops and grows in postnatal cattle at the genomic level and may imply future strategies for improving muscle growth and meat quality in cattle.

## 1. Introduction

As the largest tissue type in the body, skeletal muscle plays a central role in movement, posture, respiration, and metabolism [1]. Beyond its physiological functions, skeletal muscle from meat-producing animals provides a major source of high-quality protein, vitamins, and minerals in the human diet [2]. Skeletal muscle development can be broadly divided into prenatal myogenesis and postnatal maturation. During the prenatal stage, which includes embryonic and fetal development, muscle formation is primarily characterized by the specification, proliferation, differentiation, and fusion of myoblasts, leading to the formation of new myofibers and a substantial increase in myofiber number. In contrast, after birth, the number of myofibers remains relatively stable [3], and muscle growth is mainly achieved through myofiber hypertrophy driven by increased protein accretion [3,4], accompanied by extensive functional, metabolic, and transcriptional maturation of existing myofibers. Several biological processes are known to contribute to postnatal muscle hypertrophy. Satellite cells, as skeletal muscle stem cells in postnatal animals, contribute to muscle growth by providing additional myonuclei through fusion with existing myofibers [5]. Meanwhile, postnatal muscle maturation is accompanied by a metabolic shift from glycolytic to oxidative metabolism [6].

Transcription factors play essential roles in coordinating the stage-specific gene expression programs underlying skeletal muscle development. The regulatory functions of several muscle-specific transcription factors during prenatal myogenesis have been extensively characterized. The four myogenic regulatory factors (MRFs), including myogenic factor 5 (MYF5), myogenic factor 6 (MYF6), myoblast determination protein 1 (MYOD1), and myogenin (MYOG) [7,8,9,10], are considered core regulators of prenatal myogenesis. MYF5 and MYOD1 are essential for early myoblast determination [8,11,12], while MYOG is required for myoblast terminal differentiation [13]. MRFs regulate target gene expression mainly through binding to E-box motifs (CANNTG) as heterodimers with E-proteins [14], and many myogenic genes contain E-box elements within their regulatory regions [15]. In addition to MRFs, the myocyte enhancer factor-2 (MEF2) family, consisting of MEF2A, MEF2B, MEF2C, and MEF2D [16], also contributes to myogenic gene regulation through interactions with MRFs [17,18]. The MEF2 factors have also been implicated in postnatal muscle growth and maintenance [19].

While considerable understanding has been gained about the transcription factors and regulatory DNA sequences that control gene expression in embryonic and fetal muscles, much less is known about the regulatory programs governing gene expression during neonatal-to-adult muscle maturation. This developmental transition likely involves extensive remodeling of transcriptional programs, during which stage-specific transcription factors may establish distinct gene expression patterns required for functional and metabolic maturation. Enhancers are key cis-regulatory elements enriched with transcription factor binding sites and play essential roles in establishing tissue- and stage-specific gene expression programs. Dynamic changes in enhancer activity and transcription factor recruitment provide an important mechanism underlying developmental transitions. Active enhancers are commonly marked by histone H3 lysine 27 acetylation (H3K27ac), a histone modification associated with accessible chromatin and active transcriptional regulation [20]. Therefore, characterizing enhancer-associated transcription factor landscapes between neonatal and adult skeletal muscles may provide insights into the regulatory mechanisms underlying postnatal muscle maturation. In this study, we aimed to identify stage-specific enhancers and candidate transcription factors associated with gene regulation in skeletal muscles of neonatal and adult cattle, which are both biologically and agriculturally important species, serving as key models for studies of ruminant biology and as major livestock animals that provide meat, milk, leather, and other products.

## 2. Materials and Methods

### 2.1. Animals and Sample Collection

Five adult Angus-cross steers, 18–20 months of age, and five neonatal male Angus-cross calves, including four 1-day-old calves and one 2-day-old calf, were used in this study. All animals were provided by the Virginia Tech Beef Center. The neonatal calves were maintained with their dams and had access to maternal colostrum during the first 1–2 days after birth. The adult steers were derived from a separate feeding study conducted at the Virginia Tech Beef Center, as described in a previous study [21]. Prior to euthanasia, the steers were fed a diet containing 38.3% cracked corn, 38.4% dried distiller grains, 21.3% dairy total mixed-ration feed refusal, 1.2% mineral mix, and 0.8% ground limestone for 108 days, and then a diet containing 39% cracked corn, 40% dried distiller grains, 18% dairy total mixed-ration feed refusal, 2% mineral mix, and 1% ground limestone for 60 more days. All animals were healthy and showed no apparent signs of disease at the time of euthanasia.

Both calves and steers were euthanized by captive bolt followed by exsanguination. Muscle samples were collected from the longissimus thoracis muscle between the 9th and 13th thoracic vertebrae (T9–T13). Visible non-muscle tissues were carefully removed using sterile surgical scissors, and the muscle samples were immediately frozen in liquid nitrogen and stored at −80 °C.

All animal-related procedures were approved by the Virginia Tech Institutional Animal Care and Use Committee (IACUC #20-169).

### 2.2. RNA Extraction

Approximately one gram of muscle sample was homogenized in Tri-reagent (catalog #R2050-1-200, ZYMO RESEARCH, Irvine, CA, USA) using a polytron homogenizer (KINEMATICA AG, Malters, Switzerland). Total RNA was extracted from the homogenate using the Direct-zol RNA Microprep Kit (catalog #R2062, ZYMO RESEARCH, Irving, CA, USA). RNA concentration and purity were determined using the Nanodrop One (Thermo Fisher Scientific, Waltham, MA, USA). RNA integrity was assessed using the Agilent 2100 Bioanalyzer (Agilent Technologies, Santa Clara, CA, USA). All RNA samples passed quality-control assessment and were considered suitable for RNA-seq and RT-qPCR analyses. The RIN values (>6.6) of RNA samples are provided in Supplemental Table S1. The same RNA sample was used for RNA-seq library construction and qPCR validation.

### 2.3. RNA-Seq

RNA-seq libraries were prepared and sequenced by Novogene Corporation (Sacramento, CA, USA). Briefly, non-stranded RNA-seq libraries were constructed using the mRNA-Seq Library Preparation V2 Kit (ABclonal, Woburn, MA, USA). The RNA-seq libraries were pooled and paired-end sequenced on an Illumina NovaSeq 6000 platform (Illumina, San Diego, CA, USA) to generate at least 6 G data per library. All RNA-seq libraries passed quality control (Supplemental Table S1).

### 2.4. RNA-Seq Data Analysis

Raw RNA-seq reads were filtered to remove low-quality sequences using Trimmomatic v0.39 [22]. Clean reads were mapped to the bovine reference genome BosTaurus9 using HISAT2 (2.0.5) [23]. Gene expression raw counts were identified using featureCounts (v1.5.0-p3) [24]. Normalization of raw counts and identification of DEGs were performed using DESeq2 [25]. The cutoffs for DEGs were adjusted *p*-value (*p*_adj_) < 0.05 and |log2fold change| ≥ 0.85. Functional enrichment analyses of DEGs, including GO and KEGG pathway analysis, were performed using clusterProfiler in R (4.8.1) [26,27].

### 2.5. RT-qPCR and Data Analysis

Reverse transcription was performed using random primers (catalog #C1181, Promega, Madison, WI, USA), IImProm-IIReverse Transcriptase (catalog #A3803, Promega, Madison, WI, USA), and conditions recommended by the manufacturer. Quantitative PCR was performed in duplicate using Fast SYBR Green Master Mix (catalog #4385612, Thermo Fisher Scientific) on a 7500 Fast Real-Time PCR system (Applied Biosystems, Waltham, MA, USA). Relative mRNA expression levels were calculated using hydroxymethylbilane synthase (HMBS) as the reference gene and the 2^^(−∆∆Ct)^ method [28].

### 2.6. ChIP-Seq Library Construction and Sequencing

Chromatin cross-linking, isolation, and fragmentation were performed as described in our previous study [29]. Chromatin immunoprecipitation was carried out using 2 μg of H3K27ac antibody (catalog #ab1791, Abcam, Waltham, MA, USA) and the iDeal ChIP-seq kit (catalog #C01010051, Denville, NJ, USA), according to the manufacturers’ instructions. ChIP-seq libraries were prepared using the NEBNext Ultra II DNA Library Prep Kit for Illumina (New England BioLabs, Ipswich, MA, USA), essentially following the manufacturer’s instructions. We prepared H3K27ac ChIP-seq libraries from muscle samples from three calves and three steers, which exceeded the two biological replicates recommended by the ENCODE ChIP-seq guidelines [30]. ChIP-seq libraries were paired-end sequenced on an Illumina NovaSeq 6000 platform at Novogene Corporation.

### 2.7. ChIP-Seq Data Analysis

Sequencing adapters and low-quality sequences were removed from raw ChIP-seq reads using Trimmomatic (0.39) [22] and SAMtools (1.9) [31]. Cleaned ChIP-seq sequences were mapped to the reference bovine genome BosTaurus9 using HISAT2 (2.2.0) [23]. ChIP-seq data quality control was performed using phantompeakqualtools [30,32]. ChIP-seq peak calling was performed using Macs3 (3.0.0b1) [33]. Reproducible peaks between two biological replicates were identified using the bash software irreproducible discovery rate (IDR) (2.0.4.2) [30,34]. Calf-specific, steer-specific, and shared ChIP-seq peaks were identified using DiffBind (v3.10.1) [35,36]. Calf-specific peaks were defined as those with FDR ≤ 0.05 and fold change (calf/steer) > 0, while steer-specific peaks were defined as those with FDR ≤ 0.05 and fold change (calf/steer) < 0. Shared peaks were defined as those with FDR > 0.05 and |foldchange| < 0.5. Motif enrichment analysis of ChIP-seq peaks was performed using Homer [37]. ChIP-seq peaks were annotated using the annotatePeak function of the ChIPseeker package (1.36.0) [38]. Super-enhancers were identified using ROSE (1.3.1) [39,40].

## 3. Results

### 3.1. Gene Expression Profile Differs Significantly Between Neonatal and Adult Muscles

**Figure 1:**
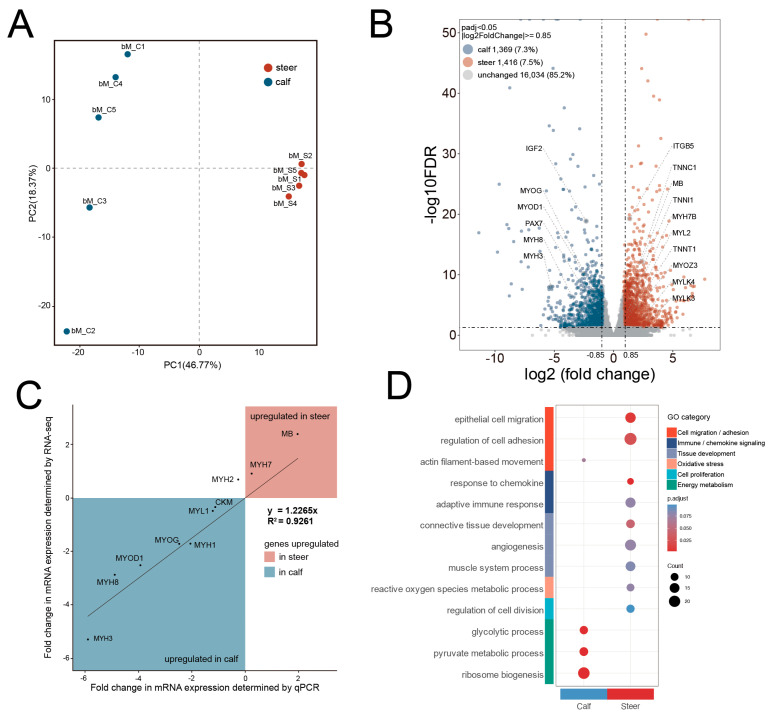
RNA-seq reveals stage-specific biological process activity in neonatal and adult muscles. (**A**) Principal component analysis (PCA) of RNA-seq data from muscle samples from five neonatal calves (bM_C1 to C5) and five adult steers (bM_S1 to S5). (**B**) Volcano plot showing numbers and percentages of genes upregulated (*p*_adj_ < 0.05 and |log2FoldChange| ≥ 0.85) in calf or steer muscle or unchanged. (**C**) Validating RNA-seq data by RT-qPCR of ten muscle-specific genes. (**D**) Gene Ontology (GO) enrichment analysis of genes upregulated in calf or steer muscle.

RNA-seq analysis of skeletal muscle samples from five newborn calves and five adult steers detected 18,819 expressed genes. Principal component analysis (PCA) revealed that gene expression profiles in neonatal and adult muscle samples differed primarily along principal component 1 (PC1), which explained 46.77% of the total variance (Figure 1A). Adult samples formed a relatively tight cluster, while neonatal samples showed greater dispersion along PC2, which explained 18.37% of the total variance (Figure 1A). A total of 2785 DEGs were identified between neonatal and adult muscles (Figure 1B). Among these DEGs, 1369 were downregulated, and 1416 upregulated from neonatal calves to adult cattle (Figure 1B). Representative genes downregulated from neonatal muscle to adult muscle included myosin heavy chain 3 (MYH3), myosin heavy chain 8 (MYH8), myoblast determination protein 1 (MYOD1), and insulin-like growth factor 2 (IGF2). Examples of genes upregulated from neonatal muscle to adult muscle included integrin beta 5 (ITGB5), myoglobin (MB), and myosin light chain kinase 4 (MYLK4).

To determine if the RNA-seq data are reliable, we measured the expression levels of ten muscle-specific genes, including MB, MYH1, MYH2, MYH3, MYH7, MYH8, MYOD1, MYOG, myosin light chain 1 (MYL1), and creatine kinase, M-type (CKM), using RT-qPCR. As shown in Figure 1C, the fold changes (neonatal/adult) in the expression levels of these genes determined by RT-qPCR were highly consistent with those determined by RNA-seq, validating the RNA-seq analysis.

### 3.2. Biological Process Activity Differs Significantly Between Neonatal and Adult Muscles

Functional enrichment analysis of DEGs between neonatal muscle and adult muscle showed that genes upregulated in neonatal calf muscle versus adult steer muscle were primarily enriched in actin filament-based movement, glycolytic process, pyruvate metabolic process, and ribosome biogenesis (Figure 1D). In contrast, genes upregulated in adult steer muscle versus neonatal calf muscle were enriched in functional categories related to regulation of cell differentiation, regulation of cell adhesion, epithelial cell migration, immune response, connective tissue development, and angiogenesis (Figure 1D). These data indicate that energy metabolism and protein translation are more active in neonatal muscle, while cell differentiation, cell migration, angiogenesis, fibrogenesis, and immune response are more active in adult muscle.

### 3.3. Identification of Active Enhancers and Transcription Factors Controlling Gene Expression in Neonatal and Adult Muscles

Active enhancers are commonly enriched for acetylation of lysine 27 on histone H3 (H3K27ac), a histone modification associated with active regulatory regions [20,41]. To identify active enhancers in skeletal muscle, we performed H3K27ac ChIP-seq on muscle samples from three newborn calves and three adult steers. Each ChIP-seq library generated at least 10 million uniquely mapped reads, with an average unique mapping rate of approximately 70% (Supplemental Table S2). Using phantompeakqualtools [32], we found that ChIP-seq data from steer muscle samples S2 and S3 and calf samples C2, C3, and C4 met the recommended quality thresholds (NSC > 1.1, RSC > 0.8) [30] (Supplemental Figure S1). Because S1 did not pass quality control, C3, C4, S2, and S3 were retained for subsequent analyses. ChIP-seq data from C2 was not included in further analyses to maintain balanced group sizes.

**Figure 2:**
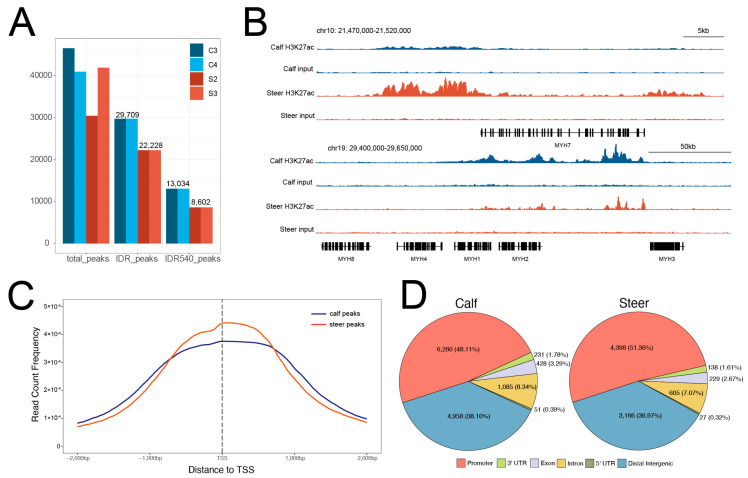
Identification of H3K27ac-marked active enhancers in neonatal calf and adult steer muscles using ChIP-seq. (**A**) Numbers of total H3K27ac ChIP-seq peaks (total_peak) identified in two calf and two steer muscle samples and reproducible peaks (IDR_peak) and high-confidence reproducible peaks (IDR540_peak) between two calf or steer samples. (**B**) Representative H3K27ac ChIP-seq peaks in calf or steer muscle. Note that these peaks are associated with several myosin heavy chain (MYH) genes. (**C**) Aggregate H3K27ac ChIP-seq signal profiles centered at transcription start sites (±2 kb). (**D**) Genomic distribution of identified H3K27ac-marked enhancers in calf and steer muscles.

In total, 46,519, 40,922, 30,432 and 41,879 H3K27ac ChIP-seq peaks were identified in calf muscle samples C3 and C4 and steer samples S2 and S3, respectively (Figure 2A). Using IDR 540 as the threshold, 13,034 and 8602 high-confidence reproducible H3K27ac ChIP-seq peaks were identified in two calf and two steer muscle samples, respectively (Figure 2A). Examples of these highly reproducible H3K27ac ChIP-seq peaks are shown in Figure 2B. Genome-wide distribution analysis indicated that H3K27ac-marked active enhancers were most concentrated in the promoter and distal intergenic regions (Figure 2C,D).

### 3.4. Stage-Specific Enhancers and Transcription Factors Regulate Distinct Aspects of Muscle Development and Function in Neonatal and Adult Cattle

**Figure 3:**
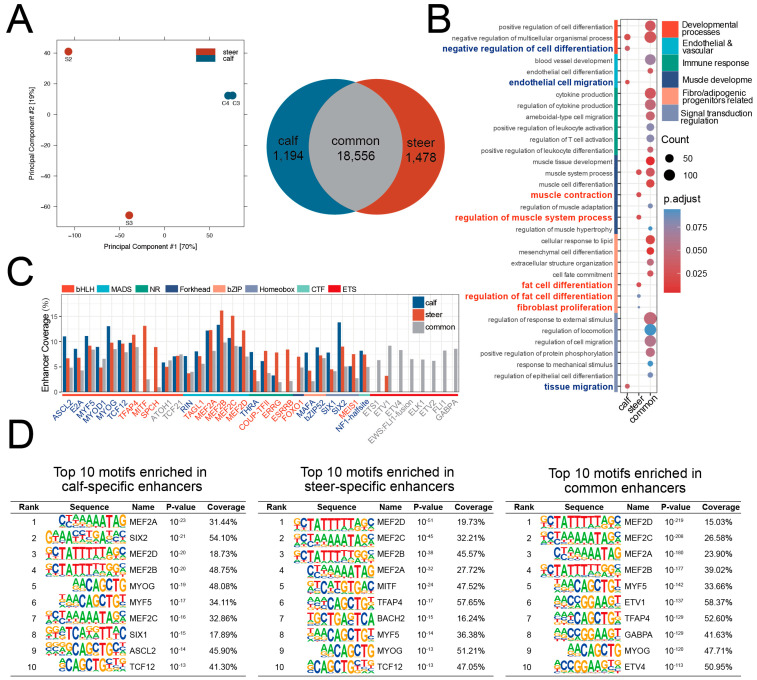
Identification of developmental stage-specific H3K27ac-marked enhancers in neonatal calf and adult steer muscles. (**A**) Principal component analysis (PCA) and Venn diagram showing calf-specific, steer-specific and common H3K27ac-marked enhancers in muscle. (**B**) Gene Ontology (GO) enrichment analysis of genes associated with calf-specific, steer-specific and common H3K27ac-marked enhancers. Several functional associations of greater interest are highlighted in blue or red. (**C**) Coverage of the top 15 motifs enriched in calf-specific, steer-specific and common H3K27ac-marked enhancers. Motifs are grouped by family and color-coded accordingly. (**D**) The top 10 motifs enriched in calf-specific, steer-specific, and common H3K27ac-marked enhancers. Motifs are ranked by *p*-value.

PCA showed clear separation of H3K27ac-marked muscle enhancers between neonatal calves and adult steers (Figure 3A). Using differential binding analysis, we identified 1194 calf-specific, 1478 steer-specific and 18,466 common H3K27ac-marked enhancers in muscle (Figure 3A). GO enrichment analysis revealed that neonatal calf-specific enhancers regulate genes functioning in tissue migration, endothelial cell migration, and negative regulation of multicellular organismal development and cell differentiation (Figure 3B). In contrast, adult steer-specific enhancers regulate genes involved in muscle system processes, muscle contraction, fat cell differentiation, and fibroblast proliferation. GO enrichment analysis revealed that H3K27ac-marked enhancers common in calf and steer muscles regulate genes functioning in many fundamental biological processes, which can be grouped into six categories: developmental processes, endothelial and vascular, immune response, muscle development, fibro/adipogenic progenitor-related pathway and signal transduction and regulation (Figure 3B).

Motif enrichment analysis predicted the top 15 motifs enriched in calf-specific active enhancers, steer-specific active enhancers, and active enhancers shared by both calf and steer muscles (Figure 3C). Among these, the homeobox family transcription factors, including SIX1 and SIX2, and the bZIP family transcription factors, including MAFA and bZIP52, showed higher motif enrichment in calf muscle, whereas members of the MADS family (MEF2A, MEF2B, MEF2C, and MEF2D), nuclear receptor (NR) family, and Forkhead family, showed increased enrichment in steer muscle compared to calf muscle (Figure 3C,D). The ETS family transcription factors showed dominant coverage in calf- and steer-shared enhancers (Figure 3C,D). Interestingly, motifs of classic myogenic bHLH transcription factors, including MYOD, MYOG, MYF5, and TCF12, showed decreased enrichment in adult muscle compared with neonatal muscle (Figure 3C,D). In contrast, other bHLH transcription factors such as TFAP4, MITF and SPCH displayed increased enrichment in adult muscle. Notably, SPCH and MITF motifs were not detected in calf muscle-specific enhancers (Figure 3C,D).

### 3.5. Integrated Enhancer–Transcriptome Analysis Identifies Key Enhancers and Transcription Factors Regulating Muscle Development and Function from Neonatal to Adult Stages

**Figure 4:**
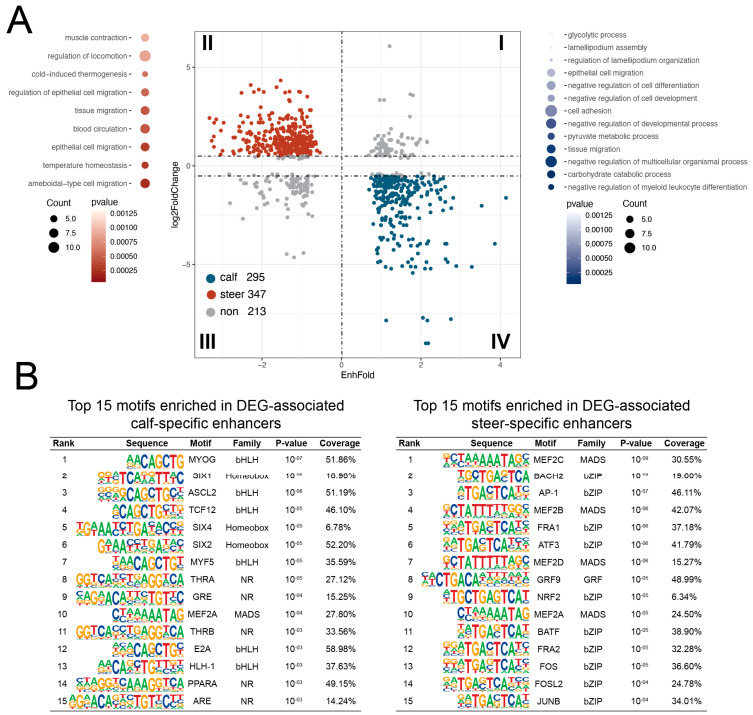
Integrative analysis of H3K27ac-marked developmental stage-specific enhancers and differentially expressed genes (DEGs) between calf and steer muscles. (**A**) Four-quadrant plot illustrating the relationship between enhancer activity changes and gene expression changes. The x-axis represents enhancer fold change (EnhFold) and the y-axis represents the corresponding gene expression change (log2FoldChange). Data points in quadrants II and IV represent putative enhancers associated with genes upregulated in calf or steer muscle. GO enrichment results are shown adjacent to quadrants II and IV, highlighting biological processes associated with steer- and calf-specific enhancer activation, respectively. (**B**) The top 15 motifs enriched in calf- or steer-specific enhancers associated with genes upregulated in calf or steer muscle, respectively. Motifs are ranked by *p*-value.

By integrating the developmental stage-specific enhancer data with the transcriptome data, we found that 295 calf-specific enhancers were associated with 195 genes upregulated in calf muscle and that 347 steer-specific enhancers were associated with 233 genes upregulated in steer muscle (Figure 4A, Supplemental Tables S3 and S4). GO enrichment analysis revealed that genes upregulated in steer muscle and associated with steer-specific enhancers were enriched in processes related to muscle contraction, regulation of locomotion, cold-induced thermogenesis, epithelial cell migration, and blood circulation, reflecting development and functional features of mature muscle (Figure 4A). In contrast, genes upregulated in calf muscle and associated with calf-specific enhancers were enriched in glycolytic process, pyruvate metabolic process, carbohydrate catabolic process, negative regulation of cell differentiation, and cell adhesion, reflecting metabolic and developmental features of immature muscle (Figure 4A).

Motif enrichment analysis showed that in calf-specific enhancers associated with genes upregulated in calf muscle, the most enriched motifs belonged to the bHLH family transcription factors, such as MYOG, TCF12, and ASCL2; the homeobox family transcription factors, such as SIX1, SIX2, and SIX4; and the NR family transcription factors, such as GR, THR, and ARE (Figure 4B). In contrast, the most enriched motifs in steer-specific enhancers associated with genes upregulated in steer muscle were the bZIP family transcription factors, such as AP-1, FOS, FRA1, and JUNB, and the MAD family transcription factors, including MEF2A, MEF2B, MEF2C, and MEF2D (Figure 4B).

### 3.6. Identification and Characterization of Super-Enhancers in Neonatal Calf and Adult Steer Muscles

**Figure 5:**
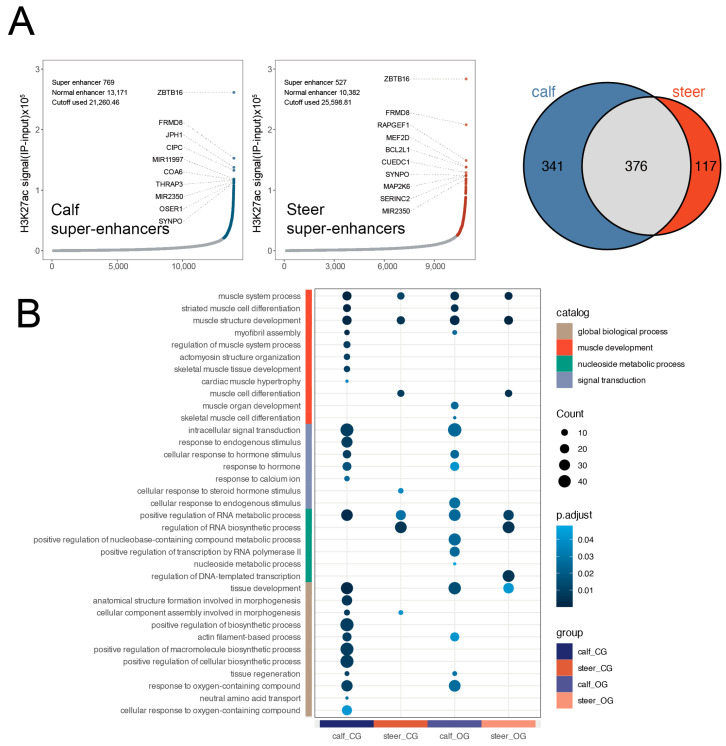
Super-enhancers (SEs) in neonatal calf and adult steer muscles control both common and distinct biological processes. (**A**) Identification of SEs in calf and steer muscles using the ROSE program (see text for details). The Venn diagram shows the numbers of SEs that are calf-specific, steer-specific, or shared between calf and steer muscles. (**B**) Functional enrichment analysis of genes potentially targeted by SEs. CG and OG on the axis refer to genes closest to and genes overlapping with calf- or steer-specific SEs.

Super-enhancers (SEs) are large regulatory genomic regions composed of clusters of enhancers with high transcription factor occupancy and strong transcriptional activity, and these elements are thought to play key roles in controlling the expression of genes involved in cell identity and major biological processes [42]. In total, 527 SEs were identified in adult steer muscle and 769 SEs in neonatal calf muscle (Figure 5A). Among them, 376 SEs were shared between the two stages, while 117 and 341 SEs were specific to steer and calf muscle, respectively (Figure 5A). Functional enrichment analysis showed that while both calf and steer muscle SEs were associated with genes participating in muscle system process, muscle structure development, and positive regulation of RNA metabolic process, they were also associated with genes functioning in distinct biological processes (Figure 5B). For example, whereas calf muscle SEs were associated with genes functioning in intracellular signal transduction, response to hormone, actin filament-based process, and response to oxygen-containing compound, steer muscle SEs were associated with genes functioning in regulation of RNA biosynthetic process and muscle cell differentiation (Figure 5B). These data indicate that SEs play an important role in the developmental and functional changes from neonatal to adult muscle.

## 4. Discussion

In this study we first profiled the transcriptomes in neonatal calf and adult steer muscles. While the PCA showed a clear separation between neonatal and adult muscle gene expression profiles, it also indicated a greater gene expression diversity in neonatal muscle than in adult muscle. A similar difference in gene expression has been reported between early-stage and late-stage porcine muscles [43]. Greater transcriptional diversity in neonatal muscle than in adult muscle probably reflects the fact that neonatal muscle is still developing and has more dynamic biological processes compared to adult muscle. Our RNA-seq data showed that the myosin heavy chain genes MYH3 and MYH8 were downregulated, whereas MYH7 was upregulated from neonatal calf to adult steer muscle. These changes indicate a transition in dominant MYH isoforms during muscle maturation and are consistent with previous findings that neonatal muscle expresses MYH3 and MYH8 as the predominant myosin heavy chain isoforms [44,45] and that adult muscle mainly expresses MYH1, MYH2, and MYH7 [46]. The downregulation of MYH3 and MYH8 from the neonatal to adult stage is consistent with their classification as embryonic and perinatal MYH isoforms, respectively [47]. Because MYH7 is the primary MYH isoform associated with slow oxidative fibers, the increased expression of MYH7 in adult steer muscle suggests that adult muscle exhibits a more oxidative metabolic profile than neonatal calf muscle [48]. Interestingly, the mRNA levels of MYH1, MYH2, and several other adult muscle genes (e.g., CKM, TNNC2, MYL1) were not markedly different between neonatal calf and adult steer muscles. This observation suggests that, from the perspective of muscle-specific gene expression, neonatal calf muscle may be more developmentally advanced than traditionally assumed. In addition, we observed downregulation of MYOD1 in adult steer muscle. This decrease may reflect a reduced proportion of satellite cells in adult muscle, consistent with the known role of MYOD1 in myoblast determination [49].

Our GO analysis showed that genes upregulated in neonatal muscle compared to adult muscle were primarily enriched in glycolytic and pyruvate metabolic processes. This suggests that mitochondrial and myoglobin levels are lower in neonatal calf muscle than in adult steer muscle, contributing to the pinker color of neonatal muscle versus the darker red color of adult muscle. Our GO analysis also indicated that ribosome biogenesis-related pathways were more enriched in neonatal calf muscle compared with adult steer muscle. This finding suggests enhanced protein synthesis activity in neonatal muscle, which is consistent with the rapid growth and maturation processes occurring during early postnatal development [50,51].

In addition to biological processes directly related to myofiber development and function, our GO analysis identified several pathways associated with the muscle microenvironment, including connective tissue development and angiogenesis. These findings may reflect coordinated remodeling of the muscle microenvironment during the transition from neonatal to adult stages. Connective tissue development may involve stromal populations, including fibro-adipogenic progenitors (FAPs) [52]. These cells have been reported to participate extensively in muscle regeneration, hypertrophy, muscle adaptation, and intramuscular fat deposition [53]. In addition, connective tissue provides the extracellular matrix framework that supports muscle growth and structural organization [54]. The enrichment of the GO term regulation of cell division may partially reflect the contribution of proliferative stromal populations during muscle remodeling. Angiogenesis is another essential biological process during muscle tissue development. Increased angiogenic activity in adult muscle may be associated with the greater proportion of slow oxidative fibers in adult muscle, as these fibers require a more substantial blood supply than fibers predominant in neonatal muscle [55]. Meanwhile, a more complex vascular network also facilitates the dynamic participation of immune cells. Correspondingly, the GO analysis identified enrichment of terms related to response to chemokine and immune adaptation in adult steer muscle. The recruitment of immune cells is known to be required for effective muscle regeneration and hypertrophy [56]. Furthermore, enhanced vascularization may improve oxygen availability and is consistent with the metabolic characteristics of oxidative muscle fibers, which may explain the enrichment of the GO term reactive oxygen species metabolic process.

In this study, we also identified tens of thousands of enhancers active in neonatal calf muscle or adult steer muscle. A substantial proportion of these enhancers were shared between the two developmental stages and enriched with binding sites for multiple members of the ETS family of transcription factors, the MEF family of transcription factors, and the bHLH family of transcription factors. These enhancers and transcription factors likely represent the core regulatory network underlying the fundamental biological processes in both neonatal and adult muscles [57,58]. A relatively small portion of these enhancers were specific to neonatal calf or adult steer muscle, and motif enrichment analysis indicated that these stage-specific enhancers were bound by both shared and stage-specific transcription factors. The neonatal muscle-specific enhancers were more enriched with binding sites for MYOG, MYF5, SIX1, SIX2, MAFA, and bZIP52, whereas the adult muscle-specific enhancers were more enriched with binding sites for MEF2A, MEF2C, MEF2D, and several nuclear receptors and Forkhead transcription factors. In other words, during muscle maturation, the chromatin coverage of classical myogenic transcription factors, including MYF5, MYOD, and MYOG [59], decreased, whereas the chromatin coverage of the traditionally associated MEF2 family transcription factors [60,61] as well as the transcription factors such as TFAP4 [29], USF2, and MITF, showed increased enrichment. These differences suggest a potential regulatory shift from early myogenic transcription factors toward MEF2, TFAP4, USF2, and MITF-dominated transcriptional control during muscle maturation. This transition aligns with known biological differences between neonatal and adult muscles as revealed by the aforementioned RNA-seq analysis: muscle development predominates in neonatal calves, whereas processes such as protein turnover, fibrogenesis, adipogenesis, and stress and immune responses become more prominent in adult muscle.

In this study, we found that many genes differentially expressed between neonatal and adult muscles were associated with stage-specific enhancers, suggesting that stage-specific enhancers play key roles in regulating differential gene expression between neonatal and adult muscles. Consistently, we found that stage-specific enhancer-associated differentially expressed genes were enriched in several biological processes uniquely active in neonatal or adult muscle. Adult muscle-specific enhancer-associated genes upregulated in adult muscle were enriched in muscle contraction and regulation of locomotion, reflecting the functional properties of mature hypertrophic muscle fibers; in cold-induced thermogenesis and temperature homeostasis, suggesting the role of mature skeletal muscle in shivering thermogenesis [62]; in blood circulation, indicating the increased oxygen demand of slow oxidative fibers in adult muscle; and in cell migration, reflecting increased tissue remodeling during muscle hypertrophy. In contrast, neonatal muscle-specific enhancer-associated genes upregulated in neonatal muscle were enriched in glycolysis and pyruvate metabolism, which are characteristic of immature muscle with a lower proportion of oxidative fibers [63].

Motif enrichment analyses of the stage-specific enhancers associated with DEGs between neonatal and adult muscles indicated a clear shift in transcription factor binding, from the bHLH transcription factors in neonatal muscle toward the MEF2 and bZIP transcription factors in adult muscle. The bHLH transcription factor family includes the classical myogenic regulators MYF5, MYOD, MRF4, and MYOG, as well as E-proteins such as TCF12, and increased binding to these transcription factors to neonatal muscle-specific enhancers is consistent with the important roles of these transcription factors in controlling satellite cell activation, myoblast differentiation, and fusion into multinucleated myofibers, processes that are highly active during prenatal and perinatal muscle development [64,65]. Increased binding of the MEF2 transcription factors, which are involved in muscle protein metabolism and structural maintenance [66,67], to adult muscle-specific enhancers is consistent with the fact that after birth, muscle development shifts toward a hypertrophic growth program, in which protein turnover gradually replaces extensive cell proliferation [68,69]. The enrichment of AP-1 binding motifs in adult stage-specific enhancers compared with neonatal stage-specific enhancers suggests a potentially important role of AP-1-related regulatory activity during muscle maturation. Although AP-1 has been widely recognized for its roles in cell proliferation, apoptosis, and differentiation [70,71,72], its functions may be context-dependent and vary across developmental stages. In adult muscle, AP-1 family members, including JUN and FOS proteins, may participate in additional regulatory processes, such as stress adaptation and immune-related responses. Consistent with this possibility, our RNA-seq analysis revealed enhanced oxidative metabolism- and immune-related biological processes in adult muscle [73], suggesting that AP-1-related regulatory programs may contribute to the establishment and maintenance of mature muscle characteristics. In addition, binding sites for NRF2, a transcription factor involved in oxidative stress responses [74], were also more enriched in adult muscle-specific enhancers than in neonatal muscle-specific enhancers. This observation is consistent with the increased oxidative capacity associated with the expansion of slow oxidative fibers during muscle maturation [2].

## 5. Limitations of the Study

This study has several limitations. First, the sample size was modest, particularly for H3K27ac ChIP-seq, which included two biological replicates per group after quality-control filtering. Although replicate concordance was assessed using the IDR framework and only reproducible peaks were retained, validation in larger independent cohorts would strengthen the findings. Second, both RNA-seq and H3K27ac ChIP-seq were performed on whole skeletal muscle tissue; therefore, the observed stage-associated differences may reflect changes in cell-type composition, cell-intrinsic regulation, or both. This is particularly relevant to pathways related to angiogenesis, immune responses, extracellular matrix remodeling, and fibroblast-associated processes.

## 6. Conclusions

The transition from neonatal to adult skeletal muscle involves extensive remodeling of both intrinsic muscle programs and the surrounding tissue microenvironment. Our integrative transcriptomic and enhancer analyses revealed that muscle maturation is accompanied by coordinated changes in biological processes related not only to myofiber development and function but also to extracellular matrix remodeling, angiogenesis, and immune- and stress-related responses, suggesting potential contributions of non-myofiber cellular components to the maturation of skeletal muscle tissue. Furthermore, stage-specific enhancer-associated transcription factor landscapes were identified between neonatal and adult muscle. Neonatal muscle enhancers were enriched for motifs associated with myogenic regulatory programs involved in early muscle development, whereas adult muscle enhancers showed increased representation of motifs linked to maturation-associated transcriptional regulation, including MEF2, AP-1, and NRF2-related programs. These findings provide novel insights into the dynamic transcription factor-enhancer regulatory networks underlying postnatal skeletal muscle maturation and identify candidate regulators for further functional investigation.

## Data Availability

The datasets generated and analyzed during the current study are available in the Gene Expression Omnibus (GEO) repository under accession number GSE329093.

## Supplementary Materials

The following supporting information can be downloaded at: https://www.mdpi.com/article/10.3390/biology15151248/s1, Supplemental Table S1: Supplemental_Table1_RNAseqQC_info.txt; Supplemental Table S2: Supplemental_Table2_lib_read_info.png; Supplemental Table S3: Supplemental_Table3_Enhgene_calf.txt; Supplemental Table S4: Supplemental_Table4_Enhgene_steer.txt; Supplemental Figure S1: Supplemental_Figure1_ChipQC.png.

## Abbreviations

The following abbreviations are used in this manuscript:

MYF5	myogenic factor 5
MYOD1	MYOD1
MYOG	Myogenin
SIX1	Sine Oculis Homeobox 1/2
MEF2(A/B/C/D)	Myocyte enhancer factor 2 (A/B/C/D)
AP1	Activator protein 1
MB	myoglobin
MHC	Myosin heavy chain
MYH(1,2,3,7,8)	Myosin heavy chain (1,2,3,7,8)
ChIP-seq	Chromatin immunoprecipitation followed by sequencing
RT-qPCR	Reverse transcription-quantitative PCR
PCA	Principal component analysis
GO	Gene ontology
FAP	Fibro-adipogenic progenitors
